# Using the Lives Saved Tool to inform global nutrition advocacy

**DOI:** 10.7189/jogh.14.04138

**Published:** 2024-08-16

**Authors:** Yvonne Tam, Yashodhara Rana, Hannah Tong, Chytanya Kompala, Jack Clift, Neff Walker

**Affiliations:** 1 Institute for International Programs, John Hopkins Bloomberg School of Public Health, Baltimore, Maryland, USA; 2 Eleanor Crook Foundation

## Abstract

**Background:**

The global nutrition community has been interested in investigating investment strategies that could be used to promote an increased focus and investment in nutrition programming in low- and middle-income countries.

**Methods:**

The Lives Saved Tool (LiST) was used to evaluate lives saved and the costs of nutrition interventions in nine high-burden countries. In this case study, we detail the analyses that were conducted with LiST and how the results were packaged to develop Nourish the Future – a five-year proposal for the US government to scale up lifesaving malnutrition interventions.

**Results:**

Scaling up a proposed package of critical nutrition interventions including micronutrient supplementation for pregnant women, breastfeeding support, Vitamin A supplementation for children, and treatments for moderate and severe acute malnutrition is an effective and cost-effective way to avert millions of child deaths and stillbirths.

**Conclusions:**

This is one of the few case studies that outlines how a nutrition modeling tool (in this case LiST) was used to engage in a prioritisation exercise to inform a US-based advocacy ask. We share reflections and provide practical insights into user motivation and preferences for existing and future modeling tool developers. This case study also emphasises how integral evidence translation and strategic advocacy are to ensure the use of the modeling results.

Malnutrition is a major cause of child deaths, contributing to about 45% of all deaths in children under five years of age. Children who are malnourished, especially those suffering from severe acute malnutrition, face an increased risk of mortality due to common childhood illnesses like diarrhea and pneumonia [1]. Those who survive suffer lifelong consequences caused by severe and irreversible cognitive and physical damage [2]. This makes addressing malnutrition critical for improving both the survival and well-being of children.

Preventing and treating malnutrition is entirely feasible, as effective solutions have long been established. Since the first Lancet series in 2008, researchers have identified high-impact nutrition interventions that are proven to save lives and improve nutrition and health outcomes for caregivers and their children [3]. Although these cost-effective solutions are at hand, they have still not been widely implemented. Less than one percent of total global development assistance is spent on high-impact nutrition interventions [4]. In fact, only 0.4 percent of US foreign assistance was allocated to prevent and treat malnutrition in 2020 [5,6]. The coronavirus disease 2019 (COVID-19) pandemic and the ongoing Ukraine crisis have further shrunk the fiscal space, making even maintaining funding for nutrition programming more difficult [7].

Against this backdrop, the world will not be able to save lives and protect the future of children without a renewed and coordinated effort. For donors especially, a tighter focus on the highest-impact interventions could reduce the complexity of intervention packages and increase the probability that coverage and impact goals are achieved. For this reason, a coalition of US-based advocacy partners led by the Eleanor Crook Foundation wanted to develop investment strategies that included the prioritisation of the most urgent and effective nutrition programmes to receive limited resources. The Lives Saved Tool (LiST) was used to conduct this analysis, since it can estimate the impact of scaling up maternal, newborn, and child health, and nutrition interventions in low- and middle-income countries (LMICs), and its main output is additional lives saved, a key metric that enables donors to understand and translate the impact of their investments. LiST has been used previously to refine national and province-level health programmes and strategies to reduce maternal and child mortality [8].

Here we present a case study on how the LiST model was used to inform US global malnutrition advocacy. While several nutrition modelling tools (including LiST) have been developed, published literature has mostly focussed on their methods and results and not enough on how the tools and the evidence they generated were applied to support policy and advocacy [9]. In this case study, we detail the analyses that were conducted with LiST and how the results were packaged to develop Nourish the Future – a five-year proposal for the US government to scale up lifesaving malnutrition interventions [10]. We also hope that the country-specific impacts and costs of scaling high-impact nutrition interventions will provide useful data for policymakers, donors, and advocates working in the nine high-burden priority countries.

## METHODS

LiST is a mathematical modelling tool that estimates the impact of improving coverage of health interventions on reducing maternal, neonatal, and child mortality and stillbirths in LMICs. General data and assumptions used in LiST and specific nutrition data and assumptions used have been described elsewhere [11]. We used Spectrum, version 6 (John Hopkins University, Baltimore, MD, USA, and Avenir Health, Glastonbury, CT, USA) for this analysis.

Briefly, LiST estimates the impact of interventions by first building a baseline for a country that includes the current levels of cause-specific mortality, levels of risk factors by age (including age-specific stunting and wasting rates), and birth outcomes (preterm and small for gestational age births). These estimates are based on the most recent data available for a country, with mortality data coming from UN global estimates; stunting and wasting rates from the most recent country-specific data on anthropometry; and coverage data from national household surveys, primarily the Demographic and Health Surveys and Multiple Indicator Cluster Surveys. LiST also includes baseline coverage of health and nutrition interventions which users can utilise to scale up the coverage of one or more interventions. Based on the efficacy of those interventions, LiST recomputes the changes in risk factors and mortality due to the increased coverage of the interventions. Long-term impacts related to lifetime productivity, such as the outcomes of additional years of schooling and lifetime earnings, were calculated based on per unit increase in height for age z-score (HAZ) among stunted children aged 12–23 months [12].

To estimate the long-term impact related to lifetime productivity, LiST first uses stunting outputs to estimate improved linear growth and quantifies education gain using a global estimate of 0.47 additional schooling years obtained per unit increase in HAZ among stunted children 12–23 months within the same birth cohort [13]. The relative gains in wages are quantified using country-specific estimates of the percent wage increase per additional year of schooling obtained [14]. Future wages are estimated using country-level gross national income (GNI) per capita, a global estimate of labour share of income (50%), and country-level labour force participation rate [15]. GNI per capita in future years is projected using an estimated annual growth rate [16], while the global estimate of the labour share of income and country-level labour force participation rate is held constant over time. Because the additional wage earnings accrue only once for each child as the birth cohort enters the labour market, the average working years is set to 44 by default, starting at the age of 16 years and retiring at the age of 60 [15]. A 3% discount rate was used to calculate the net present value of future wages.

In the case of costs, LiST uses an ingredients-based approach to estimating intervention costs based primarily on costs for supplies, equipment, and drugs for the population in need being covered by the scale-up of interventions. It also estimates program and systems costs in addition to the direct programme costs. More details on the overall costing approach are available elsewhere [17].

For this analysis, we used an iterative process to utilise LiST to select and refine the package of interventions and the coverage scenario to quantify the impact and investment needed for the Nourish the Future proposal. The analysis focussed on nine LMICs (Burkina Faso, Chad, Democratic Republic of Congo (DRC), Ethiopia, Madagascar, Mali, Niger, Nigeria, and Pakistan), all of which have large mortality burdens due to malnutrition and were maternal and child health priority countries for the US Agency for International Development (USAID).

Broadly speaking we followed a five-step process to conduct and package this analysis.

First, while LiST includes more than 70 maternal, newborn, and child health, and nutrition interventions, we focussed on the 14 interventions [18–36] that reduce mortality due to malnutrition via reducing maternal anaemia [37,38], low birth weight [39], stunting, wasting, [40] and diarrhoea morbidity [41]. The interventions also improved lifetime productivity due to a reduction in stunting [13].

Second, we narrowed down the list of 14 interventions to those that have existing large-scale delivery platforms that could be scaled up to increase coverage rates to full coverage. This was an important consideration since the Nourish the Future proposal had a five-year time horizon (2021–25) which meant there was limited time to develop new platforms.

Third, we used the missed opportunity tool in LiST to assess the relative impact of remaining nutrition interventions by additional lives saved for all nine countries. The missed opportunity tool scales up each intervention in LiST from their current to universal coverage, one at a time and in isolation from one another [42]. Next, we considered incremental intervention costs to rank cost-effective nutrition interventions.

Fourth, for the selected package of interventions, we modelled multiple coverage scenarios with targets at 65%, 75%, 85%, and 95%, and at a mixed coverage scenario to compare the potential impact and costs. We eventually used the mixed coverage scenario, where we scaled up the coverage of nutrition interventions in different packages from current coverage in 2020 to 95% in 2021 and maintained until 2025. Interventions that were considered improbable to reach such high coverage were scaled to 50% instead. Coverage of other maternal, newborn, and child health interventions not included in the package was kept constant at current coverage from 2021 to 2025. We selected these coverage targets since the purpose of this exercise was to estimate the impact of a potential strategy to reduce malnutrition for advocacy (Table S1 and S2 in the **Online Supplementary Document**).

Finally, we packaged the results to inform the health systems proposal for Nourish the Future, which included the selected interventions and the number of child deaths that can be averted by scaling them, as well as estimates of rate reductions for under-five mortality, stillbirths, stunting, and wasting, along with additional years of schooling and lifetime earnings gained due to a reduction in stunting. This was done to inform advocacy efforts that showed long-term impact on countries' progress. Lastly, we estimated overall financing needs to inform specific asks from policymakers and donors.

## RESULTS

### Selection of priority package of interventions

LiST includes 14 nutrition interventions that have an established evidence base to reduce malnutrition (**Table 1**). From them, we excluded balanced energy-protein supplementation for pregnant women and zinc supplementation for children as there are currently no platforms at scale for these two nutrition interventions [43].

**Table 1:** List of interventions in the LiST with impact on reducing malnutrition

Continuum of care	Interventions in LiST with impact on reducing malnutrition
Periconceptual	Folic acid fortification
	Iron fortification
Pregnancy	Balanced energy supplementation
	Calcium supplementation
	Iron supplementation in pregnancy
	Multiple micronutrient supplementation in pregnancy
Preventive	Appropriate complementary feeding
	Breastfeeding promotion
	Vitamin A supplementation
	Zinc supplementation
Treatment	Treatment for moderate acute malnutrition
	Treatment for severe acute malnutrition
	Vitamin A for the treatment of measles
	Zinc for treatment of diarrhea

LiST – Lives Saved Tool

**Figure 1:**
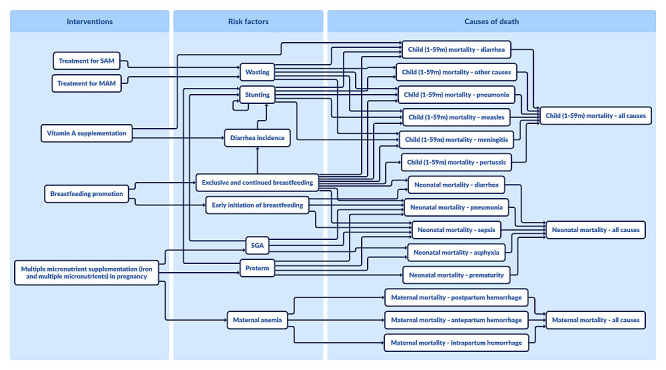
The Power 4 – multiple micronutrient supplementation in pregnancy, breastfeeding promotion, Vitamin A supplementation, and treatment of moderate and severe acute malnutrition.

Next, we conducted the missed opportunity analysis for the remaining 12 interventions. The results allow one to compare the impact of each intervention, and rank top-performing interventions that often are highly effective at reducing a main cause of death or risk factor in the country but have low current coverage. Multiple micronutrient supplementation in pregnancy (MMS), breastfeeding promotion (BFP), Vitamin A supplementation (VAS), and treatment of moderate acute malnutrition (MAM) and severe acute malnutrition (SAM) ranked favourably for averting deaths and stillbirths with low incremental costs. This package of interventions, dubbed the ‘Power 4,’ has proven effects in reducing child mortality and stillbirths (**Figure 1**).

### Results of scaling the Power 4 package to the mixed coverage scenario

On scaling up MMS, BFP, and VAS to 95% coverage, and MAM and SAM to 50% coverage, we found that the selected package could reach more than 177 million pregnant women with MMS, support more than 76 million mothers with breastfeeding support, provide Vitamin A supplementation for nearly 122 million children, and treat 32 million children suffering from wasting (**Table 2**).

**Table 2:** Additional number of services provided, and the population reached by the Power 4

Country	Mothers reached with multiple micronutrient supplementation (2021–25)	Mothers supported with breastfeeding (2021–25)	Children provided Vitamin A supplementation (2021–25)	Children treated for wasting (2021–25)
Burkina Faso	5 352 700	2 934 200	747 600	1 371 300
Chad	4 541 100	3 461 800	4 279 800	1 077 800
DRC	24 647 800	9 187 500	17 764 800	3 674 200
Ethiopia	24 192 100	8 028 900	39 057 500	4 611 300
Madagascar	5 488 800	2 237 600	419 200	1 684 900
Mali	5 705 000	2 689 200	14 754 000	1 129 000
Niger	7 590 700	4 442 000	8 575 600	2 122 800
Nigeria	52 360 300	28 200 600	30 648 500	10 347 500
Pakistan	47 264 200	14 397 400	5 474 800	5 597 200
Total	177 142 600	75 579 200	121 721 800	31 616 000

DRC – Democratic Republic of Congo

In total, this package could avert a combined 1.2 million deaths and stillbirths over five years (2021–25). Comparing rates in 2025 relative to 2020, this package could result in an average of 8.4% reduction in under five mortality rate and 8.5% reduction in stillbirth rate. Scaling up the Power 4 would cost as low as an average of USD 1600 per child death and stillbirth averted in the DRC and as high as an average of USD 4300 per child death and stillbirth averted in Madagascar (**Table 3** and **Table 4**).

**Table 3:** Mortality impact and costs of the Power 4

Country	Number of stillbirths averted, and child lives saved (2021–25)	Total incremental intervention costs in USD (2021–25)	Costs in USD (average 2021–25) per stillbirth and under-five mortality death averted
Burkina Faso	31 200	87 017 700	2800
Chad	45 800	85 408 900	1900
DRC	167 100	263 024 300	1600
Ethiopia	115 900	313 091 000	2700
Madagascar	25 600	109 671 400	4300
Mali	39 700	82 509 200	2100
Niger	51 800	118 637 400	2300
Nigeria	478 100	988 655 700	2100
Pakistan	216 200	502 037 800	2300
Total	1 171 400	2 550 053 400	-

DRC – Democratic Republic of Congo

**Table 4:** Mortality rate reductions of the Power 4

	Under-five mortality rates (deaths per 1000 live births)		
**Country**	**2020**	**2025**	**Percentage of reduction in 2025 relative to 2020**
Burkina Faso	87.5	81.3	−7.2
Chad	113.8	102.6	−9.9
DRC	84.8	78.1	−7.9
Ethiopia	50.7	46.4	−8.5
Madagascar	50.6	46.1	−8.8
Mali	94.0	86.3	−8.2
Niger	80.4	73.0	−9.2
Nigeria	117.2	106.4	−9.2
Pakistan	67.2	62.5	−7.0
Average			−8.4
	**Stillbirth rate (stillbirths per 1000 total births)**		
**Country**	**2020**	**2025**	**Percentage of reduction in 2025 relative to 2020**
Burkina Faso	19.5	17.8	−8.53
Chad	27.5	25.1	−8.55
DRC	27.2	24.9	−8.56
Ethiopia	24.6	22.5	−8.53
Madagascar	16.5	15.1	−8.56
Mali	19.7	18.0	−8.53
Niger	19.6	17.9	−8.54
Nigeria	22.3	20.3	−8.58
Pakistan	30.6	28.0	−8.55
Average			−8.55

DRC – Democratic Republic of Congo

In the longer term, it is estimated to avert 3.2 million child-years of stunting in the same time frame; as a result, the package would add 313 300 years of additional schooling and USD 754 million in total lifetime earnings (**Table 5**). It is further estimated to produce a 1.8% and 42.0% reduction in stunting and wasting prevalence rate (less than –2 standard deviations) for children 6–59 months old (**Table 6**).

**Table 5:** Long-term impact of the Power 4

Country	Total child-years of stunting averted	Total additional years of schooling for children with one unit increase in HAZ	Total lifetime earnings in USD for children with one unit increase in HAZ
Burkina Faso	41 400	1200	2 823 100
Chad	86 500	11 800	10 424 300
DRC	336 900	29 800	27 227 700
Ethiopia	683 800	89 900	196 815 300
Madagascar	78 500	5300	8 618 300
Mali	135 900	15 800	19 749 100
Niger	177 600	21 000	38 865 700
Nigeria	1 077 700	102 600	270 298 200
Pakistan	557 700	35 900	179 164 400
Total	3 176 100	313 300	753 986 200

DRC – Democratic Republic of Congo, HAZ – height-for-age Z score

**Table 6:** Stunting and wasting rate reductions of the Power 4

	Global stunting (less than −2 SD) rate for 6–59 m		
**Country**	**2020**	**2025**	**Percentage of reduction in 2025 relative to 2020**
Burkina Faso	37.5	37.2	−0.9
Chad	43.2	42.5	−1.8
DRC	45.8	45.2	−1.2
Ethiopia	41.8	40.7	−2.6
Madagascar	53.1	52.6	−1.0
Mali	32.8	31.7	−3.1
Niger	47.0	46.0	−2.0
Nigeria	46.0	45.1	−1.9
Pakistan	39.2	38.6	−1.6
Average			−1.8
	**Global wasting (less than −2 SD) rate for 6–59 m**		
**Country**	**2020**	**2025**	**Percentage of reduction in 2025 relative to 2020**
Burkina Faso	14.5	8.4	−42.02
Chad	13.2	7.6	−42.03
DRC	7.6	4.4	−41.98
Ethiopia	9.5	5.5	−41.98
Madagascar	14.3	8.3	−41.97
Mali	13.5	7.8	−41.99
Niger	18.3	10.6	−42.00
Nigeria	10.5	6.1	−42.02
Pakistan	6.2	3.6	−41.95
Average			−41.99

DRC – Democratic Republic of Congo, m – months, SD – standard deviation

### Dissemination and advocacy of the Power 4 priority package in Nourish the Future

Finally, we packaged the results in Nourish the Future – a five-year plan for the US Government to scale up the most lifesaving nutrition interventions in the hardest-hit countries. The proposal emphasises two pillars of change – health and food systems – that must work in harmony for impacts to be sustainable. The results of this analysis informed specific programme recommendations and financing needs for strengthening health systems across USAID-priority countries to deliver improved nutrition. Advocates used Nourish the Future in education and engagement efforts with decision-makers across the executive and legislative branches of government. Since the publication of Nourish the Future in 2021, the US Government has taken some steps to elevate high-impact malnutrition interventions, including the Power 4. The US Global Food Security Strategy identifies the Power 4 interventions as high-priority health system complements to US food and agricultural efforts [44]. The Global Malnutrition Prevention and Treatment Act of 2021 reinforces the need to focus on high-impact health systems interventions as part of the US Government’s comprehensive malnutrition response. In 2022, USAID announced a USD 200 million investment to the United Nations Children’s Fund (UNICEF) to scale up wasting treatment in 15 crisis-hit countries [45]; in 2024, the USAID committed an additional USD 200 million to wasting treatment.

## DISCUSSION

Nutrition modelling tools have been employed to furnish evidence, facilitate planning, and bolster advocacy efforts across a range of contexts within LMICs [9]. This is one of the few case studies that outline how a nutrition modelling tool (in this case LiST) could be used to engage in a prioritisation exercise to inform a US-based advocacy ask. Considering that not all interventions can be fully implemented given increasing budgetary constraints, focussing on a prioritised list of effective interventions can contribute to achieving the desired results. The COVID-19 pandemic and the ongoing Ukraine crisis have further shrunk the fiscal space, making even maintaining funding for nutrition programming more difficult. Against this backdrop, it is more important than ever to have a tighter focus on the highest-impact interventions that can reduce the complexity of intervention packages and increase the probability that coverage and impact goals are achieved.

Nourish the Future, which included the modelling results, was released and used as an advocacy tool coinciding with the start of the Biden administration when policy decisions were being made [10]. The results of this analysis informed specific programme recommendations and financing needs for strengthening health systems across USAID priority countries to deliver improved nutrition. US-based advocacy organisations deployed Nourish the Future as a tool and strategic asset in education and engagement opportunities with US government decision-makers. For example, in the autumn of 2021, the Eleanor Crook Foundation and the leaders from over 20 international non-governmental organisations and philanthropies met with USAID Administrator Samantha Power to discuss Nourish the Future, describing the opportunity for USAID to usher in a new era of global health by scaling up proven ways to address global malnutrition.

Core components of the proposal were reflected in the US Government’s released Global Food Security Strategy in the autumn of 2021. The final strategy included many of the recommendations contained in Nourish the Future related to food systems and nutrition, alongside a special call-out on the importance of the Power 4 interventions. Since the publication of Nourish the Future in 2021, USAID has also announced a USD 200 million investment to UNICEF to scale up wasting treatment in 15 crisis-hit countries [45].

Some limitations should be noted with this analysis. First, it was conducted in 2020, so the evidence base was considered until that point in time. Since then, the evidence base for certain nutrition interventions like small-quantity lipid-based nutrient supplements has strengthened [46]. Second, the analysis includes only costs related to the delivery of the nutrition intervention and not the total costs. For instance, in the case of Vitamin A supplementation, the modelled cost would include the cost of the supplement as well as some fraction of the costs paid to health workers to deliver the intervention; it would not, for instance, include the cost of the warehouse to store the tablets. This is not likely a significant limitation, since the same delivery platform of care is used to administer multiple health and nutrition interventions so their costs would not be limited to only one intervention. Third, as the timeframe of this analysis coincided with the COVID-19 pandemic, much remains to be seen from primary data if the health system and health service delivery experience disruptions due to various mitigation strategies to combat the pandemic. Any disruptions could have also changed the population in need of intervention and their micronutrient deficiency statuses. With a lack of data to support the change in coverage of interventions due to the pandemic, we did not assume coverage improvement of other nutrition interventions, or other health interventions included in LiST. Should their coverage increase despite the disruptions, their inclusion will reduce the impact attributed to the selected package of nutrition interventions, as many of these other interventions reduce the same risk factors and causes of death. Similarly, should their coverage decrease due to the disruptions, the impact attributed to the selected package will increase.

Using LiST was advantageous for several reasons. First, it allows for the estimation of ‘lives saved,’ which is a key metric that enables donors to understand and translate the impact of their investments [47]. To our knowledge, LiST is the only tool using this metric as its main output. Second, LiST is continually updated, thereby providing the latest data to conduct analyses, including nutrition-specific updates and expansions implemented as advised by an external advisory group [48]. While static analyses such as the Investment Framework for Nutrition and the Lancet Series provide a list of high-priority nutrition interventions and the magnitude of expected impact, LiST provides a real-time tool with updated data on impacts and costs. Like any other model, the quality of modelling outputs is highly dependent on the quality of modelling inputs. Users should interpret these lives saved while considering the quality of the main modelling inputs used to establish the baseline of the country and the change in coverage of interventions, as we described in the methods section above. Third, the modelling tool makes it easy to model different scale-up scenarios and conduct sensitivity analyses to key assumptions. This feature is critical to allow programme implementers to adjust different constraints to weigh opportunity costs and engage in a co-creation process. Lastly, LiST helps users understand the various pathways through which impact is achieved which helps increase understanding among users, advocates, or policymakers, which then further increases the likelihood of the results being used. These features of LiST are integral in generating confidence and facilitating ease of use even among users who may not be accustomed to using modelling tools.

Further, LiST is a widely used and well-reputed modelling tool that has been published in peer-reviewed journals and is therefore considered to have strong credibility. This feature has been instrumental in generating buy-in for the results of Nourish the Future among a range of stakeholders such as policymakers, programme implementers, and advocates. These reflections add to an emerging body of work that is unpacking why users select different modelling tools [9]. We also hope that our reflections provide practical insights into user motivation and preferences for existing and future modelling tool developers.

Our case study also outlines how integral evidence translation and strategic advocacy are to ensuring that the modelling results are utilised in practice. Results from this analysis, coupled with programmatic recommendations, were translated into simple and specific asks to policymakers. For example, using the data from the analysis, the Nourish the Future proposal succinctly summarised what needs to be prioritised for programming, as well as why and at what cost, which are key dimensions of concerns for policymakers. Multiple dissemination activities were conducted including bilateral meetings, public events, and speeches to disseminate the findings to a broad network of stakeholders, but also use the findings to make specific asks of stakeholders. Lastly, our experience also highlights the importance of targeting evidence translation with key policy cycle stages. In this case, the proposal was developed in early 2021 to coincide with the entry of the Biden administration.

## CONCLUSIONS

This case study outlines how a nutrition modelling tool (in this case, LiST) was used to engage in a prioritisation exercise to inform a US-based advocacy ask. It also emphasised how integral evidence translation and strategic advocacy are to ensuring the use of data-driven, evidence-based modelling results. We hope the shared reflections and practical insights will motivate the development of additional use cases of modelling to inform global health advocacy. Additional and continual documentation of how technical knowledge can be packaged to inform evidence-based nutrition programs, policies, and action, especially at the country level, will be needed to increase focus and investment for multi-sectoral involvement in improving nutrition programming in LMICs.

## Additional material


Online Supplementary Document
