# Supplementary material for: Using the Lives Saved Tool to inform global nutrition advocacy
Source: J Glob Health. 2024 Aug 16;14:04138. doi: 10.7189/jogh.14.04138 (PMC11327894; doi:10.7189/jogh.14.04138)
Supplement: Online Supplementary Document [file jogh-14-04138-s001.pdf]

APPENDIX

Table S1. Baseline and Target coverage of The Power 4 interventions used in the analysis

Country	Intervention	Baseline coverage 2020 (%)	Target coverage 2021-2025(%)
Burkina Faso	Iron supplementation in pregnancy	50.2	0.0
Burkina Faso	Multiple micronutrient supplementation in pregnancy	0.0	95.0
Burkina Faso	Promotion of breastfeeding	22.1	95.0
Burkina Faso	Vitamin A supplementation	99.0	99.0
Burkina Faso	SAM - treatment for severe acute malnutrition	26.1	50.0
Burkina Faso	MAM - treatment for moderate acute malnutrition	0.0	50.0
Chad	Iron supplementation in pregnancy	11.0	0.0
Chad	Multiple micronutrient supplementation in pregnancy	0.0	95.0
Chad	Promotion of breastfeeding	0.3	95.0
Chad	Vitamin A supplementation	70.0	95.0
Chad	SAM - treatment for severe acute malnutrition	18.3	50.0
Chad	MAM - treatment for moderate acute malnutrition	0.0	50.0
DRC	Iron supplementation in pregnancy	4.7	0.0
DRC	Multiple micronutrient supplementation in pregnancy	0.0	95.0
DRC	Promotion of breastfeeding	51.5	95.0
DRC	Vitamin A supplementation	78.0	95.0
DRC	SAM - treatment for severe acute malnutrition	11.2	50.0
DRC	MAM - treatment for moderate acute malnutrition	0.0	50.0
Ethiopia	Iron supplementation in pregnancy	5.1	0.0
Ethiopia	Multiple micronutrient supplementation in pregnancy	0.0	95.0
Ethiopia	Promotion of breastfeeding	53.7	95.0
Ethiopia	Vitamin A supplementation	48.0	95.0
Ethiopia	SAM - treatment for severe acute malnutrition	12.0	50.0
Ethiopia	MAM - treatment for moderate acute malnutrition	0.0	50.0
Madagascar	Iron supplementation in pregnancy	7.6	0.0
Madagascar	Multiple micronutrient supplementation in pregnancy	0.0	95.0
Madagascar	Promotion of breastfeeding	45.4	95.0
Madagascar	Vitamin A supplementation	96.0	96.0
Madagascar	SAM - treatment for severe acute malnutrition	1.4	50.0
Madagascar	MAM - treatment for moderate acute malnutrition	0.0	50.0

Mali	Iron supplementation in pregnancy	28.0	0.0
Mali	Multiple micronutrient supplementation in pregnancy	0.0	95.0
Mali	Promotion of breastfeeding	34.7	95.0
Mali	Vitamin A supplementation	9.0	95.0
Mali	SAM - treatment for severe acute malnutrition	41.8	50.0
Mali	MAM - treatment for moderate acute malnutrition	0.0	50.0
Niger	Iron supplementation in pregnancy	28.6	0.0
Niger	Multiple micronutrient supplementation in pregnancy	0.0	95.0
Niger	Promotion of breastfeeding	21.7	95.0
Niger	Vitamin A supplementation	64.0	95.0
Niger	SAM - treatment for severe acute malnutrition	40.0	50.0
Niger	MAM - treatment for moderate acute malnutrition	0.0	50.0
Nigeria	Iron supplementation in pregnancy	30.5	0.0
Nigeria	Multiple micronutrient supplementation in pregnancy	0.0	95.0
Nigeria	Promotion of breastfeeding	26.6	95.0
Nigeria	Vitamin A supplementation	80.0	95.0
Nigeria	SAM - treatment for severe acute malnutrition	5.8	50.0
Nigeria	MAM - treatment for moderate acute malnutrition	0.0	50.0
Pakistan	Iron supplementation in pregnancy	29.4	0.0
Pakistan	Multiple micronutrient supplementation in pregnancy	0.0	95.0
Pakistan	Promotion of breastfeeding	46.6	95.0
Pakistan	Vitamin A supplementation	92.0	95.0
Pakistan	SAM - treatment for severe acute malnutrition	0.0	50.0
Pakistan	MAM - treatment for moderate acute malnutrition	0.0	50.0

Table S2. Source of coverage for The Power 4 interventions used in the analysis

The Power 4 interventions	Coverage source	Notes
Multiple micronutrient supplementation in pregnancy	Household survey- DHS	Baseline coverage reflects iron supplementation for pregnancy from the latest household survey. The baseline coverage of multiple micronutrient supplementation in pregnancy for all countries is 0%.
Breastfeeding promotion	Household survey- DHS	Used proxy of the percent of children 1-5 months of age that are exclusively breastfed.
Vitamin A supplementation	UNICEF	
MAM - treatment for moderate acute malnutrition	Not available	Coverage data for this indicator are not typically available. Currently set at 0 for baseline
SAM - treatment for severe acute malnutrition	UNICEF	Coverage estimates calculated from:<br><br>The UNICEF Global SAM Management Update Tool (NutriDash): 2012 – 2016. Available at www.acutemalnutrition.org.<br><br>The UNICEF-WHO-The World Bank Group Joint Child Malnutrition Estimates: 2012 – 2016. Available at www.acutemalnutrition.org.
